# Repurposing of Yunnan Baiyao as an Alternative Therapy for Minor Recurrent Aphthous Stomatitis

**DOI:** 10.1155/2012/284620

**Published:** 2012-12-04

**Authors:** Xiaosong Liu, Xiaobing Guan, Ruiyang Chen, Hong Hua, Yang Liu, Zhimin Yan

**Affiliations:** ^1^Department of Oral Medicine, School and Hospital of Stomatology, Peking University, Beijing 100081, China; ^2^Department of Periodontics & Oral Medicine, Beijing Stomatological Hospital, Capital Medical University, Beijing 100050, China; ^3^Department of Oral Medicine, Tianjin Stomatological Hospital, Nankai University, Tianjin 300041, China

## Abstract

The study was designed to evaluate the efficacy and safety of an herbal extract of Yunnan Baiyao formulated in toothpaste as an alternative therapy for minor RAS. A randomized, double-blind, placebo-controlled clinical trial (from March 2010 to March 2011) was conducted on a cohort of 227 minor RAS patients. The toothpaste containing Yunnan Baiyao was used twice daily as part of the patient's routine oral hygiene for 5 days. An assessment of ulcerative size and pain was recorded on day 0 (baseline), day 3, and day 5. Any noted adverse reactions were recorded. All data were analyzed using the SAS software 8.0. As a result, the toothpaste containing Yunnan Baiyao began to present noticeable effectiveness on ulcer healing (ulcer size) by day 3 (27.5% versus 15.8%, *P* < 0.05), which further improved by day 5 when compared to the placebo (66.4% versus 50.0%, *P* = 0.01). A significant difference in alleviating pain was noted on day 5 for those who used the toothpaste containing Yunnan Baiyao (66.4% versus 51.8%, *P* < 0.05). No side effects were noted as a result of the Yunnan Baiyao. Therefore, Yunnan Baiyao may provide an alternative therapy for minor ulcers by promoting healing.

## 1. Introduction

Recurrent aphthous stomatitis (RAS) is a common oral disorder with prevalence of 25% [1], characterized by recurrent ulcers on unkeratinized oral mucosa. Three clinical types were classified as minor, major, and herpetiform [2]. Minor aphthous represents 80–85% of RAS, presenting with 3–10 mm painful ulcers in diameter up to 5 lesions concurrently and usually lasting for 10–14 days each [3]. Currently, there is no definitive curative treatment for RAS, given the unclear etiology, but the consensus recommendation is to lessen the pain and duration of ulcers by suppressing the local immune response and preventing secondary infection [1]. Therefore, topical agents including corticosteroids, antimicrobials, and analgesics make up the first choice for RAS patients due to the minimum serious adverse effects [4]. Topical corticosteroids such as triamcinolone acetonide, prednisolone, and betamethasone [5] are the major remedies that are available and have helped in reducing local inflammation and hastening the healing process. Chlorhexidine and tetracyclines, being antimicrobials, provide another local anti-inflammatory approach for RAS [1]. However, with the prolonged and frequent exposure to certain topical corticosteroids or antimicrobials, relevant drug resistance, oral flora imbalance, and secondary fungal infection may lead to the prescription of more potent therapeutic agents and even systemic administration [6]. Consequently, more potential risks will arise for health such as adrenal suppression, myelosuppression, and even inducing neoplasm. 

In this study, we suggest that an herbal medicine approach may offer an alternative therapy for minor RAS. Yunnan Baiyao is a well-known traditional Chinese medicine, formulated in a powder or capsule form. It was initially and widely used in wounds for its antihemorrhagic hemostatic function [7, 8] and further in gastrointestinal bleeding. Yunnan Baiyao powder has been generally applied on RAS among Chinese population [9]. However, because of the absence of reliable clinical data, together with relevant non-English publications, there is limited widespread recognition on its clinical utility. In this study, we conducted a randomized, double-blind, placebo-controlled clinical trial that included 227 patients with minor RAS to assess its efficacy and safety when formulated in toothpaste. 

## 2. Materials and Methods

### 2.1. Ethical Approval

Ethical approval for this study was obtained by Peking University Institutional Review Board. Each patient agreed and signed the informed consent prior to the study. The study design and protocol were in accordance with both the Helsinki Declaration and related Chinese regulatory laws in reference to conducting clinical trials.

### 2.2. Participants

A randomized, double-blind, placebo-controlled study was performed in three stomatology clinical centers: School and Hospital of Stomatology, Peking University (Peking Center); Stomatological Hospital, Capital Medical University (Capital Center); Stomatological Hospital, Nankai University (Nankai Center). A total of 240 patients were enrolled in the study. The breakdown from the sites is as follows: 84, 78, and 78 patients were from Peking, Capital, and Nankai Centers, respectively. Patients enrolled into the study had a verified history of at least two episodes of RAS in the past 12 months without any definitive cause. Below are the inclusion and exclusion criteria.

Inclusion criteria: (1) both male and female aged 18 to 65 years old; (2) patients diagnosed as minor recurrent aphthous stomatitis with the duration of each ulcer in excess of 5 days; (3) fresh ulcers available with less than 72 hours eruption.

Exclusion criteria: (1) hypersensitive to various medical agents; (2) concurrent acute infectious disease; (3) pregnancy or lactation; (4) concurrent other immunology disorders; (5) accepting systemic administration of corticosteroids or immunosuppressive agents within 3 months; (6) aphthous-like ulcers related to certain systemic disorders such as ulcerative colitis, Crohn's disease, Behçet's syndrome, and serious anemia; (7) aphthous-like ulcers related to drug such as nonsteroidal anti-inflammatory drugs (NSAIDs) and antihistamines; (8) accepting anaesthetic therapy within 24 hours, systemic antibiotics within 2 weeks, or other management for oral ulcers within 72 hours prior to the study; (9) neoplasm patients; (10) volunteers of other clinical trials on medical agents or toothpaste within one month.

### 2.3. Materials

Experimental toothpaste (120 grams weight prepackaged; offered by Yunnan Baiyao Group Co., Ltd., China) contained 0.65% (about 0.78 gram) of the active extract from Yunnan Baiyao, along with sodium phosphate dibasic dehydrate, sorbitol, hydrated silica, sodium lauryl sulfate, flavoring essence, pectin, and sodium benzoate. Placebo toothpaste (120 grams weight prepackaged; offered by Yunnan Baiyao Group Co., Ltd., China) contained the above ingredients, except for the active Yunnan Baiyao extract, though it exhibited similar color, odor, and flavor to the experimental toothpaste.

### 2.4. Randomization

Both experiment and placebo toothpastes (1 : 1 allocation ratio) were randomized using a computer-based random number generator and distributed to the centers. The randomized list was sealed, and its contents were recorded in the protocol which was conserved by the third party (an assigned statistical company). Both patients and investigators were blinded until the end of study.

### 2.5. Study Intervention

The patients were instructed to brush the teeth twice daily (in the morning after getting up and before going to bed) for 5 days, using 1 gram of toothpaste provided (equivocal to approximately 6-7 lines on a standard toothbrush) for 3 minutes, while other analogues were forbidden. The ulcer size and pain level were measured and recorded at the respective stomatology clinics on days 0 (baseline), 3, and 5 by two assigned independent clinical investigators. Any oral mucosal complications and side effects during the study as a result of the toothpaste, as well as body temperature, pulse, respiration, and blood pressure, were recorded at the time of the visit, accompanied by a detailed oral examination. If the ulcer disappeared within the 5-day study period, the experimental and/or placebo toothpaste was collected. 

Prior to the study, all assigned clinicians from the different clinical centers were trained by the principal investigator as to the standard operating procedures, which included measuring the ulcers, conducting the visual analog scale (VAS), and recording/documenting the data. To calibrate the values measured by different investigators, a kappa statistical analysis was performed on ten patients (*κ* = 0.8). A standard brushing duration was enforced, and calibrated timers were given to the participants of the study. The study was standardized in reference to toothpaste consumption, where 2 grams were recommended per day for each patient, and patients who used less than 80% or more than 120% of the 2 grams per day of toothpaste were removed from the study. The daily consumption of the toothpaste was calculated by weighing the remainder of the toothpaste at the end of the study.

### 2.6. Clinical Evaluation

The fresh ulcers, as described as being developed within 72 hours of onset and must be clearly visible and accessible to the investigators, were documented. The assessment of the surface area of the ulcer was measured in millimeters by a dental probe (Shanghai Dental Instrument Factory, China). Ulcer size was assessed as the product of maximum diameter and its vertical diameter. Pain intensity was measured using a VAS, where the amount of pain recorded ranged from 0 (no pain) to 10 (unbearable pain). Pain was assessed by irritating the ulcer with the periodontal probe. The values were collected by the assigned investigators.

The efficacy index (EI) was measured as a function of either ulcer size index (EI size) or pain intensity index (EI_pain_), calculated as follows:

(1) EI(size)=E0−(E3/E5)E0×100%,EI(pain)=E0−(E3/E5)E0×100%.

*E*0, *E*3, and *E*5 represent the respective data values (either based on ulcer size or pain) that were collected on days 0, 3, and 5. The response to therapy (experimental versus placebo) was broken down to four criteria (Table 1) (ClinicalTrials.gov registration number: NCT01652625).

### 2.7. Statistical Analysis

Parametric and nonparametric statistical tests were used for the analysis of comparing the ulcer size or pain scale between experimental and placebo groups. The *t-*test was used in the analyses of ulcer size, *Wilcoxon rank-sum* test for categorical data of VAS (pain), and *chi-square* test for the comparison of gender and efficacy index (EI). All data were analyzed using the SAS software 8.0 (SAS Institute Inc., USA). *P* < 0.05 was considered statistically significant. 

## 3. Results

A total of 240 patients were enrolled into the study (from March 2010 to March 2011), and after the start of the study, thirteen patients withdrew due to personal reasons, leaving a total of 227 patients. The patients were randomly placed into one of two groups, experimental (113 patients) or placebo (114 patients) group. At the initial visit (day 0-baseline), no differences were noted based on age, gender, duration of previous ulcers, existing ulcer size, and pain intensity (Table 2). The 227 patients enrolled had compliant for the 5-day study period. 

### 3.1. Effect of Yunnan Baiyao on Size of Ulcer

**Figure 1:**
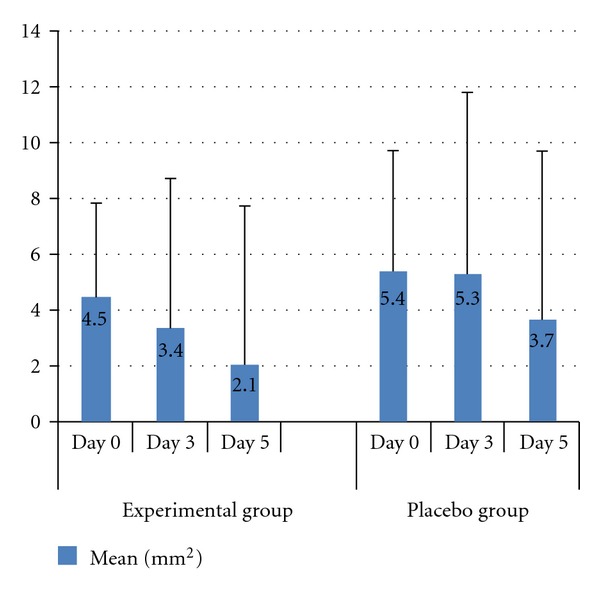
A comparison of ulcer size between experimental and placebo groups for day 0, day 3, and day 5.

Observations on the effect of Yunnan Baiyao on ulcer size were assessed for both experimental and placebo groups during day 3 and day 5 using the efficacy index (EI) as described in Section 2. Table 2 provides a summary of these results. On day 3, starting with those that were in the EI_size_ = 100% category, 11 and 6 from the experimental group and placebo group, respectively, were healed fully. In the EI_size_ = 70–100% category, 20 and 12 from the experimental group and placebo group, respectively, had moderate improvement. If we paired EI = 100% and EI = 70–100% categories as considered significant improvement of ulcer and EI = 0–30% and EI = 30–70% as nonsignificant improvement, an obvious between-group difference was identified in the rate of significant improvement relative to the size of the ulcer, as displayed by 27.4% in the experimental group compared to 15.8% in the placebo group (*P* < 0.05). On day 5, 39 patients from the experimental group (34.5%, including the 11 from day 3) fell into the EI_size_ = 100% category, and 31.9% (36/113) of patients fell into the EI_size_ = 70–100%, whereas in the placebo group, 21.5% (24/114) and 28.9% (33/114) fell into the EI_size_= 100% and EI_size_ = 70–100% categories, respectively. Consequently, a relatively higher rate of significant improvement was exhibited in experimental group than in placebo group (66.4% in experimental group versus 50.0% in placebo group, *P* = 0.01). Meanwhile, the between-group difference in reference to average size of ulcers was displayed on day 3 (3.4 mm^2^ versus 5.3 mm^2^, *P* = 0.01) and further on day 5 (2.1 mm^2^ versus 3.7 mm^2^, *P* < 0.01) (see Table 3 and Figure 1). The results suggested a potential effectiveness of the active extract from Yunnan Baiyao on reducing the size of ulcer.

### 3.2. Effect of Yunnan Baiyao on the Levels of Pain

The level of pain was recorded using the VAS for both groups. The measurement was performed on 113 experimental patients and 114 placebo patients. Based on the EI criteria for pain, by day 3, the VAS values have decreased by 24.8% (28/113) from the experimental group for both EI_pain_ = 100% and EI_pain_ = 70–100% categories, as compared to 14.9% (17/114) in the placebo group. By day 5, the VAS values had further decreased for majority of the patients for both EI_pain_ = 70–100% and EI_pain_ = 100% categories, 66.4% (75/113) and 51.8% (59/114) for the experimental and placebo groups, respectively. An obvious difference was shown by day 5 but not by day 3 (see Table 4).

There were no pathological changes related to body temperature, blood pressure, pulse, and respiration as a result of the study. We noted two patients from placebo group complained of lingual numbness (not related to the study) though, after further followup, they recovered without any medical intervention.

## 4. Discussion

In this study, we examined the repurposing of a Chinese herbal medicine to alleviate RAS in terms of its efficacy and safety. Yunnan Baiyao is a combination of compounds from a variety of Chinese medical herbs. It has been known to be effective for its unique antihemorrhagic hemostatic function/capacities in the Chinese population [7, 8]. For nearly a century, the recipe remains protected by Chinese government due to intellectual property concerns.

A total of 13 milligrams of the Yunnan Baiyao extract is present in 2 grams of the toothpaste that was designed for adult minor RAS patients in the present paper. By weighing the rest of the toothpaste at the end of the study, the permitted toothpaste consumption was confined to 1.6–2.4 grams daily (within 80–120% of 2 grams). Consequently, there was 0.8 gram difference at most between the minimum and maximum allowed amount, containing 5.2 milligrams of Yunnan Baiyao, in different patients. Along with the similar amount of toothpaste consumption, the standardized brushing approach and brushing duration instructed by the investigators prior to the study, together with the ulcer site accessible to the foam from brushing, were warranted to the same amount of foam from brushing presenting per unit of area of the ulcers. Future studies will be needed to evaluate the dose/responses and titrations of Yunnan Baiyao based on toothpaste amount. The results presented in the study were reliable due to the between-group similarity of ulcer size and ulcer pain level on initial investigation and the duration of ulcers within 72 hours onset. 

As a result of the study, by day 3, the effect of Yunnan Baiyao began to display on reducing ulcer size compared to the placebo, as a noticeable decrease on the average size of the ulcer, and by day 5, the size was insignificant. Additionally, by day 3, and day 5, the surface area of ulcer closed by no less than 70% in larger proportion of the patients with Yunnan Baiyao toothpaste than that with the placebo. Apart from the active extract of Yunnan Baiyao, there was no difference between experimental and placebo toothpastes in both ingredients and amount of the components. Therefore, it was suggested that Yunnan Baiyao active extract might play an important role in reference to ulcer healing. Yunnan Baiyao formulated in toothpaste could be recommended as an adjunctive treatment of minor RAS or may even be effective for patients with severe RAS in conjunction with other remedies. The effect of Yunnan Baiyao on ulcer healing based on our study will give impetus to utilizing another approach of a carrier of the extract that would be focused (increased concentration) at the sight of the ulcer which includes an ointment approach or a delivery as in the form of a gel or mouthwash. However, just the toothpaste formulation of Yunnan Baiyao, might hinder more patients (27.4% and 66.4% of the patients on days 3 and 5, resp.) in the present study getting a significant improvement on ulcer size. The foam from brushing teeth limited the duration of Yunnan Baiyao extract contacting with the surface of lesions, and subsequently the penetration towards subepithelial layer infiltrated with amounts of inflammatory cells. With the closing of more ulcers, pain from the ulcers disappeared. The effectiveness of Yunnan Baiyao toothpaste on pain, exhibited in the present study on day 5, might be the result of healing of ulcer, but not directly related to the analgesic function.

RAS is an etiologically complex disorder and considered to be the common manifestation of a group of disorders with different causes, rather than a single entity [10]. Immune mechanism including local immune may involve in the development of ulcers, which was supported by the histological infiltration of neutrophils, lymphocytes, and plasma cells in the epithelium affected [11]. Various cytokines may contribute to the pathogenesis of ulcers [12], as elevated IL-2, IFN-*γ*, and TNF-*α*, while lower concentrations of IL-10 were reported in lesions of RAS patients [13, 14]. Although the exact mechanism is unknown, the effect of Yunnan Baiyao on ulcer size in our study might be attributable to its role in the anti-inflammatory or immunosuppressive pathways, hence accelerating the healing process. The property of Yunnan Baiyao in promoting intestinal mucosal cell spreading, confirmed by more extensive lamellipodia at the leading edges of the wounds [15], may benefit the healing of oral ulcers starting from the periphery. Moreover, the immunosuppressive activity of Yunnan Baiyao was demonstrated by a highly selective cytotoxicity towards B and T lymphocytes and an inhibitory ability on the expressions of TNF-*α* and IFN-*γ* in colonic mucosa and serum of experimental colitis mice, discovered lately by Li and his colleagues [15]. The upregulated level of TNF-*α* in oral lesions was considered to play an important role in RAS eruption, which is produced by gamma-delta T cells [16], macrophages, and mast cells [17] and may induce inflammation by its chemotactic action on neutrophils [18]. Thus, as a final therapeutic strategy, a group of anti-TNF-*α* agents were employed by oral clinicians for severe and refractory RAS patients, including pentoxifylline, thalidomide, adalimumab, and infliximab [1] and eventually obtained therapeutic success in shortening the duration of ulcer and lengthening the free ulcer course. Therefore, Yunnan Baiyao may be a potential alternative anti-TNF-*α* agent for RAS.

Ulcers in RAS patients were generally exposed to plenty of microorganisms which can induce and promote the inflammatory reaction. Protecting the lesions against microbes may benefit the healing of these ulcers. Although the antimicrobial activity of Yunnan Baiyao on inflammatory bowel disease was denied by Li et al. for its ineffectiveness on *E. coli* growth [15], the effect of Yunnan Baiyao on RAS-related microbes was not detected in this study. 

In our previous results (not shown), 21.6 mg/kg/day of Yunnan Baiyao active extract was administered intragastrically in Sprague-Dawley rats (SD rats) (170–210 grams weight) for a duration of 30 days. The extract was diluted into 2 milliliters of water and provided to the rats by rat stomach-cleaning apparatus. Based on the human equivalent dose (HED) conversion formula recommended by FDA [19], 13 milligrams of Yunnan Baiyao extract permitted daily in present study was far more less than the HED of 222 milligrams for an adult patient weighing 70 kilograms. The safety evaluation of Yunnan Baiyao on adult RAS patients, whose oral mucosa interacts with a maximum dose of 13 milligrams of Yunnan Baiyao for a total of 6 minutes daily for 5 days, was assessed by monitoring body temperature, blood pressure, pulse, and respiration, as well as any side effects. The absence of pathological changes of the above values observed in the present paper suggested the safety of Yunnan Baiyao for topical use as a toothpaste carrier. The lingual numbness noted by few patients in the placebo group may be as a result of the common constituents of toothpaste, but not related to the Yunnan Baiyao extract.

## 5. Conclusion

In conclusion, the repurposing of Yunnan Baiyao may have a beneficial effect on healing of minor RAS by short-term topical application without significant side effects, which was demonstrated by our present randomized, double-blind, placebo-controlled clinical trial.

## Figures and Tables

**Table 1:** The criteria of response to therapy.

Criteria	Definition
EI = 100%	Either ulcer healed or pain disappeared
EI = 70–100%	More than 70% of either ulcer surface area closed or VAS value
	decreased, including 70% but not 100%
EI = 30–70%	More than 30% of either ulcer surface area closed or VAS value
	decreased, including 30% but not 70%
EI = 0–30%	Less than 30% of either ulcer surface area closed or VAS value
	decreased

**Table 2:** The baseline demography data of the patients.

	Experimental group (*n* = 113)	Placebo group (*n* = 114)	*P *
Age (year) (mean ± SD)*	31.9 ± 10.9	31.4 ± 11.8	0.75
Gender**			0.17
Male	22 (19.5%)	31 (27.2%)	
Female	91 (80.5%）)	83 (72.8%)	
Duration of previous ulcer (day)*	9.1 ± 3.0	9.4 ± 3.8	0.98
Ulcer size (mm^2^) (mean ± SD)*	4.5 ± 3.3	5.4 ± 4.3	0.21
Ulcer pain (VAS)***			0.21
0	4	1	
1	8	6	
2	9	11	
3	23	25	
4	16	9	
5	27	23	
6	9	16	
7	9	14	
8	5	7	
9	2	1	
10	1	1	

VAS: visual analog scale.

*: *t*-test was used in analysis of quantitative data, *P* > 0.05.

**: *chi-square* test was used in analysis of enumeration data, *P* > 0.05.

***: *Wilcoxon rank-sum *test was used in analysis of categorical data, *P* > 0.05.

**Table 3:** The number of patients in experimental and placebo groups on day 3 and day 5 based on ulcer size.

	Day 3			Day 5		
			*P *			*P *
	Experimental group (%)	Placebo group (%)		Experimental group (%)	Placebo group (%)	
Number	113	114		113	114	
Size (mean ± SD)	3.4 ± 5.3	5.3 ± 6.5	0.01*	2.1 ± 5.6	3.7 ± 6.0	<0.01**
Significant improvement	31 (27.4)	18 (15.8)	0.03***	75 (66.4)	57 (50.0)	0.01****
EI_size_ = 100%	11 (9.7)	6 (5.3)		39 (34.5)	24 (21.1)	
EI_size_ = 70–100%	20 (17.7)	12 (10.5)		36 (31.9)	33 (28.9)	
Nonsignificant improvement	82 (72.6)	96 (84.2)		38 (33.6)	57 (50.0)	
EI_size_ = 30–70%	35 (31.0)	38 (33.3)		16 (14.2)	21 (18.4)	
EI_size_ = 0–30%	47 (41.6)	58 (50.9)		22 (19.5)	36 (31.6)	

*and **: *P *values represent the comparisons of ulcer size between groups on days 3 and 5, respectively.

***and ****:* P* values represent the comparisons of significant improvement and nonsignificant improvement on days 3 and 5, respectively.

( ): it represents percentage per group.

**Table 4:** The number of patients in the placebo and experimental groups on day 3 and day 5 based on the level of pain.

	Day 3			Day 5		
			*P *			*P *
	Experimental group (%)	Placebo group (%)		Experimental group (%)	Placebo group (%)	
Number	113	114		113	114	
Significant improvement	28 (24.8)	17 (14.9)	>0.05*	75 (66.4)	59 (51.8)	<0.05**
EI_pain_ = 100%	16 (14.2)	6 (5.3)		48 (42.5)	45 (39.5)	
EI_pain_ = 70–100%	12 (10.6)	11 (9.6)		27 (23.9)	14 (12.3)	
Nonsignificant improvement	85 (75.2)	97 (85.1)		38 (33.6)	55 (48.2)	
EI_pain_ = 30–70%	34 (30.1)	43 (37.7)		24 (21.2)	32 (28.1)	
EI_pain_ = 0–30%	51 (45.1)	54 (47.4)		14 (12.4)	23 (20.2)	

*and **: *P* values represent the comparisons of significant improvement between groups on days 3 and 5, respectively.
